# Human Milk Vitamin B3 (Niacin and Vitamer) Concentrations: Secondary Analysis of a Randomized, Placebo-Controlled Trial of Maternal Nicotinamide Supplementation in Rural Tanzania

**DOI:** 10.1016/j.ajcnut.2026.101397

**Published:** 2026-06-17

**Authors:** Mark D. DeBoer, Joann M. McDermid, Daniela Hampel, Setareh Shahab-Ferdows, Gabrielle Mazzoni, Janet M. Peerson, Sarah E. Elwood, Rebecca J. Scharf, Samwel Jatosh, James A. Platts-Mills, Estomih Mduma, Lindsay H. Allen

**Affiliations:** Department of Pediatrics, University of Virginia, Charlottesville, VA, USA; Division of Infectious Diseases & International Health, University of Virginia, Charlottesville, VA, USA; Institute for Global Nutrition, University of California, Davis, CA, USA; Institute for Global Nutrition, University of California, Davis, CA, USA; Department of Pediatrics, University of Virginia, Charlottesville, VA, USA; U.S. Department of Agriculture, Davis, CA, USA; Division of Infectious Diseases & International Health, University of Virginia, Charlottesville, VA, USA; Department of Pediatrics, University of Virginia, Charlottesville, VA, USA; Division of Infectious Diseases & International Health, University of Virginia, Charlottesville, VA, USA; Haydom Global Health Research Centre, Haydom Lutheran Hospital, Haydom, Tanzania; Division of Infectious Diseases & International Health, University of Virginia, Charlottesville, VA, USA; Haydom Global Health Research Centre, Haydom Lutheran Hospital, Haydom, Tanzania; U.S. Department of Agriculture, Davis, CA, USA

**Keywords:** vitamin B3, nicotinamide, niacin, human milk, breast feeding

## Abstract

**Background:**

The risk of vitamin B3 (niacin) deficiency is increased if maize is consumed without improving bioavailability during food preparation and with limited consumption of niacin-rich foods.

**Objectives:**

We assessed human-milk vitamin B3 concentrations and their relationship to child outcomes as a secondary analysis of an RCT of nicotinamide supplementation to lactating mothers in rural Tanzania.

**Methods:**

The ELICIT study, conducted in Haydom, Tanzania, randomized lactating females to supplemental nicotinamide (250 mg/d vs. placebo) from study entry (infant age <2 weeks) until 6-months. Among 1173 mother/child dyads, human-milk nicotinamide and B3 vitamer concentrations at lactation months 1 and 5 were compared to international reference limits (5^th^ and 95^th^ percentile) from the recent MILQ study, and child anthropometry and cognitive development (Malawi Developmental Assessment Tool, MDAT) relationships were assessed.

**Results:**

The proportion of human-milk total vitamin B3 concentrations below the lower reference limit increased over time in both those without and with nicotinamide supplementation (2.9% vs. 0.5% ≤0.720mg/L at 1 month; 28.8% vs. 9.9% ≤0.549mg/L at 5 months). Maternal nicotinamide supplementation was associated with many participants having human-milk total vitamin B3 concentrations exceeding the upper reference limit (72.9% ≥3.284mg/L at 1 month; 50.7% ≥2.760mg/L at 5 months), and a sustained higher median concentration with supplementation vs. placebo (by 291% at month 1 and 281% at month 5). No associations were observed between human-milk total vitamin B3 or vitamer concentrations and child weight, height or head circumference up to 18 months or with MDAT scores at 18 months.

**Conclusions:**

Without maternal nicotinamide supplementation, lactating females had a greater prevalence of human-milk total vitamin B3 concentrations below the 5^th^ percentile reference limit. Although supplementation was associated with greater total vitamin B3 and vitamer concentrations, there was no association between human-milk B3 and child growth or development outcomes up to 18 months.

**Registry:**

ClinicalTrials.gov (NCT03268902)

## INTRODUCTION

Vitamin B3 includes three main forms or vitamers, including niacin or nicotinic acid, nicotinamide or niacinamide, and nicotinamide riboside, with all forms converted into nicotinamide adenine dinucleotide (NAD). It is an essential micronutrient that must be consumed in the diet or derived endogenously from tryptophan (1). Deficiencies range from severe, manifesting as overt pellagra, to under-recognized sequelae such as birth defects (2). Dietary deficiency is observed in regions, including sub-Saharan Africa, where maize is the main staple food in a diet that is not diversified with niacin-rich food sources such as animal, poultry, fish or fortified foods (3, 4). While maize contains many nutrients, including niacin and the precursor amino acid tryptophan, bioavailability is reduced without nixtamalization (soaking in alkaline solution) prior to cooking that is not traditionally practiced in sub-Saharan Africa and many other regions (1, 5).

Malawi is a region with a high reported maize consumption (Malawi average=356 grams/person/day vs global average=48grams/person/day). Maternal supplementation with a lipid-based supplement with 20 mg/d niacin equivalents (NE) was successful in increasing human milk nicotinamide concentrations (430 to 566 μg/L at 2 to 6 weeks; 219 to 281 μg/L at 24 weeks of lactation) among Malawian females despite few consuming adequate dietary niacin intakes (6). In the rural region surrounding Haydom, Tanzania, food and nutrition insecurity is common and associated with household poverty and deficits in childhood growth and acute malnutrition (7). Maize is a key staple food for lactating females and children, and rich sources of niacin including meat, fish, poultry, or fortified foods, are typically consumed on a limited basis. Consequently, maternal and child undernutrition is common, with childhood stunting reaching 70% in a previous study in this region (8).

As part of a randomized clinical trial (9), lactating females were randomized to receive nicotinamide supplementation (250 mg/d nicotinamide, with U.S. RDA being 17 mg among lactating females) or placebo during the first 6 months of lactation (10). The current analysis was focused on assessing:1) rural Tanzanian human milk total vitamin B3 concentrations by nicotinamide treatment group over time, 2) comparing these concentrations to internationally-derived reference ranges (13); 2) 3) assessing the relationship between human milk total vitamin B3 concentrations and child anthropometry and developmental outcomes. We hypothesized that human milk vitamin B3 concentrations among females in the placebo study arm would be lower than a healthy reference range (13), and below the vitamin B3 concentrations observed in the nicotinamide supplementation group. We also hypothesized that greater human milk B3 concentrations would be associated with longer-term improvements in child anthropometry and developmental outcomes.

## METHODS

We conducted a secondary longitudinal substudy of the Early Life Interventions for Childhood Growth and Development in Tanzania (ELICIT) trial. The study design, participant characteristics, and growth and cognitive outcomes of the ELICIT study have been reported previously (9, 11, 14). Briefly, this was a 2×2 factorial randomized controlled trial assessing the effects of two different interventions (daily maternal nicotinamide supplementation followed by daily child nicotinamide supplementation; scheduled child antimicrobials) on childhood growth and cognitive development age 0 to 18 months, with a primary outcome of child length-for-age z-score at 18 months (9, 11). The primary results demonstrated no associations with either length-for-age Z-scores (LAZ) or cognition (9, 11, 14). The protocol was approved by the National Institute for Medical Research of Tanzania, the Tanzanian Medicine and Medical Device Authority and the Institutional Review Board at the University of Virginia. Study oversight was provided by FHI360 (North Carolina, USA). Females provided written informed consent to participate during pregnancy or at the time of enrollment. Inclusion criteria at enrollment were maternal age ≥18 years, infant age <14 days, and stated intent to reside within a 25-km radius of Haydom Lutheran Hospital for the study duration. Exclusion criteria were multiple gestation pregnancy, significant birth defect or neonatal illness, infant weight <1500 g at enrollment and lack of stated intent to breastfeed. Recruitment occurred between September 5, 2017 and August 31, 2018 and study procedures and data collection continued through February 18. 2020. The study attained its target enrollment of 1188 mother-infant pairs, which was calculated based on our ability to determine a difference in LAZ of 0.176 at 18 months between intervention groups (9, 11). The study was registered at ClinicalTrials.gov (NCT03268902). The current report is a secondary analysis of vitamin B3 vitamin and vitamer concentrations in human milk samples from nursing mothers in the trial.

### Interventions

As part of the factorial design, approximately four equal groups of participants received either: 1) both interventions (maternal followed by child nicotinamide plus child antimicrobials), 2) placebo for both interventions; 3) maternal followed by child nicotinamide plus child placebo for antimicrobial intervention, or 4) maternal followed by child placebo for nicotinamide intervention plus child antimicrobials. For this study, these four groups were combined into two for analyses, either receiving maternal nicotinamide or placebo. For the nicotinamide intervention, lactating mothers received a nicotinamide 250 mg tablet (or placebo) daily (with U.S. RDA being 17 mg among lactating females) during months 0 to 5.99 (Supplementary Figure 1). A maternal dose of 250 mg of nicotinamide was based on a desire to raise total human milk nicotinamide over the 6-month period to deliver an amount that approached but did not exceed the 2000 μg/d recommended by the National Academy of Medicine reference Adequate Intake for infants from birth to 6-months (10). We based these calculations on a study of Malawian females who received nicotinamide equivalent doses of 20 mg daily and had human milk levels of nicotinamide assessed at 24 weeks (6). These calculations took into account nicotinamide concentrations in human milk among those unsupplemented and a milk consumption volume of 0.8 L/d. Using this approach and a maternal dose of 250 mg, we estimated that at no point would infants receive greater than 2000 μg/d in nicotinamide. At 6 months of age children then received sachets of 100 mg nicotinamide powder (or placebo) mixed in their own food daily from age 6 to 18 months (with U.S. RDA being 4–6 mg among children in this age range). Adherence was monitored through monthly questionnaire and every 2-month counting of pills/sachets. Nicotinamide (tablets and sachets) and the corresponding placebos were manufactured by Vita-gen (New York, USA).

For the child antimicrobial intervention, participants received azithromycin or the corresponding placebo (both manufactured by Universal Corp, Kenya) as a single oral dose of 20 mg/kg of 200 mg/5 mL suspension at months 6, 9, 12, and 15. Nitazoxanide or placebo (both provided by Romark, Florida, USA) was administered as a 3-day course of 100 mg twice daily of 100 mg/5 mL suspension at months 12 and 15.

### Data collection

Study visits occurred at the participants’ home, both for screening and enrollment and then every month until 18 months. Child length, weight, and head circumference were measured at enrollment and every three months until 18 months. Child length was assessed using calibrated measuring length boards, weight using digital scales, and head circumference using a measuring tape (9, 11). All child anthropometry measures were converted to WHO z-scores, including length-for-age (LAZ), weight-for-age (WAZ) and head-circumference-for-age (HCZ). Maternal height was measured using a standing stadiometer and weight was measured with a digital scale at approximately 11-months postpartum.

At all visits, mothers responded to questionnaires that focused on lactation status and child health status. These data were by maternal report and only hospitalizations of the infant were verified by study staff. Exclusive breastfeeding was defined using WHO criteria as an infant receiving only human milk and no other food or drink (including water)(15). At the initial monthly visit, mothers also responded to questions regarding household demographic, social and economic factors. Socioeconomic status was assessed using a scoring system based on improved water and sanitation, assets, maternal education and household income (WAMI)(16) developed for use in low-resource settings.

### Cognitive Development Assessment

At the 18-month study visit, trained personnel administered the Malawi Development Assessment Tool (MDAT), a cognitive scale created to assess child development in children living in a rural sub-Saharan context. The MDAT is an assessment with good reliability in sub-Saharan Africa (94–100%)(17). Additional details on the use of this scale in ELICIT have been published previously (12). Briefly, MDAT is a neurodevelopmental scale for use in early childhood, that includes the following subscores: fine motor, gross motor, language, and social. The ELICIT cognitive team worked to adapt, pilot, and validate the MDAT locally for Haydom. The items, words, and tasks used in the assessment were similar to those developed for the original study environment in rural Malawi. Six field workers completed four weeks of training and practice in MDAT implimentation. Items were scored as 1 (pass) or 0 (not pass). Within each MDAT subtest, children completed items until they received a score of 0 on each item for six items in a row. Standardized MDAT Z-scores were calculated using version 1.1 of the MDAT Scoring Application.

### Collection and Measurement of Human milk Vitamin B3 Concentration

Human milk samples were collected at 1 and 5 months. Mothers cleansed the breast around the areola with soap and water, rinsing with deionized water. Approximately 8 mL of human milk was hand-expressed into a wide-mouthed sterile container mid-feed. The container was then placed on ice before being transported to the laboratory, where human milk was aliquoted and kept at −80 degrees until time of analysis.

Human milk vitamin B3, vitamers and tryptophan were analyzed using an ExionLC AD coupled to a 6500+ QTRAP mass spectrometer (Sciex, Framingham, MA). Briefly, 100uL of whole milk was defatted, deproteinized with methanol, and evaporated. The dried extract was reconstituted in 10mM aqueous ammonium formate and filtered prior to analysis using the LC-MS system as previously described (18). The LC-MS system is highly sensitive for detection, with standard curve ranges and signal -to-noise ratios for individual vitamers as provided in Supplementary Table 1. Samples were analyzed in singlicate with study time points (months 1 and 5) tested separately. Inter-day variation was established using the QC sample. Each prepared set included 24 samples comprising 22 study and 2 QC samples, which were analyzed first and last of each set as biological replicates. A typical analytical run included 4 sets which included 8 QC samples as biological replicates. The QC inter-day variations for all analytes throughout the analyses across all visits are provided in Supplementary Table 1. Values below the standard curve range (considered lower limit of quantification, LLoQ) were included in calculations as being just below the LLoQ, while levels above this range were computed as measured. Total vitamin B3 in human milk was calculated by adding the molar amounts of each of the individual vitamers, which were converted to nicotinamide equivalents (as mg/L) from the individual vitamer levels using the following equation: vitamin B3 (mg/L nicotinamide equivalents) = nicotinamide + (122.12 × nicotinamide adenine dinucleotide /663.43) + (122.12 × nicotinamide mononucleotide/334.22) + (122.12 × nicotinamide riboside /255.25).

### Statistical analysis

Statistical analyses were performed using SAS version 9.4 (Cary, NC). For assessing differences between study intervention groups, statistical significance was assessed using *t*-tests. If concentrations were non-normally distributed, data were log-transformed prior to statistical analyses and entering in regression models. Inter-correlations between individual B3 vitamers, B3 and tryptophan were assessed to determine Pearson’s *r* values by intervention group and time point to assess correlations of B3 concentrations between lactation months 1 and 5. To facilitate direct comparison of effect sizes between lactation time points in assessing long-term associations between B3 concentrations and outcomes, standardized values were used to generate cohort-specific z-scores (mean= 0, standard deviation= 1) for all regression analyses. To characterize these factors prior to the primary analyses (assessing correlations with growth and cognitive development), multiple potential relationships between factors, including inter-factor correlations, differences according to intervention sub-group, sex-based differences, and associations with WAMI were considered. In assessing potential outcomes related to human milk vitamin B3 concentrations, linear regression was used to assess relationships between human milk total vitamin B3 concentration at both 1 and 5 months with child anthropometry (LAZ, WAZ and HCZ) at subsequent time points and also relationships between human milk total vitamin B3 concentration and scores on the MDAT (both total score and individual component score). In keeping with WHO recommendations, no LAZ, WAZ or HCZ values were beyond 6 or −6. Because intervention subgroups did not differ in these outcomes (as reported previously(11, 12)), both intervention subgroups were combined for the current analysis to assess potential relationships with human-milk B3 concentrations specifically. These assessments were performed using unadjusted analysis and models adjusted for sex and WAMI. The categories for each covariate used in the regression analyses were: 1. human milk analytes: total B3 concentration (cohort-based standardized score with mean 0 and standard deviation 1); 2. sociodemographic: infant sex (male/female), WAMI (socioeconomic status index (16), described above; potential range is from 0–1); 3. anthropometry: LAZ, WAZ, HCZ (all WHO age-based standardized scores with mean 0 and standard deviation 1.0); 4. MDAT developmental scales: Overall, Fine Motor, Gross Motor, Language, Social (all standardized for this cohort, with mean 0 and standard deviation 1.0). As a sensitivity analysis, we also assessed whether there was a difference in these outcomes when comparing the infants where human milk B3 concentrations were below the reference range 5^th^ percentile vs. those with concentrations above the 95^th^ percentile. Statistical significance for the regression was determined using a hierarchical false discovery rate (FDR) approach. For each block of outcomes (e.g., three anthropometry outcomes—LAZ, WAZ and HCZ, separately for each time point or five cognitive development outcomes), *P* values were ranked in order, smallest to largest, *P*[1] < *P*[2] < *P*[3]…. < *P*[5]. For each *P* value, statistical significance was determined by computing 0.05*i/x, where × was the number of outcomes. Thus, in the case of the five cognitive outcomes significance for *P*[1] was set at 0.05*1/5 = 0.01; *P*[2] was set at 0.05*2/5 = 0.02, etc. Then the first *P* value where *P*[i] exceeded its calculated significance threshold value was identified, and all *P* values prior to this were declared ‘significant by false discovery rate (FDR)’ (19).

## RESULTS

We assessed human milk vitamin B3, vitamer and tryptophan concentrations from 1173 participants, including 1163 participants at 1 month of lactation and 1136 participants at 5 months, with concentrations analyzed at both time points for 1126 participants. Maternal characteristics are shown in Table 1. Participating mothers had a low average education duration with only a mean of 6.4 years and a low family socioeconomic status with median monthly income US$12.90. The median number of months of exclusive breastfeeding was 5 months (interquartile range 5, 6). As reported previously, there was a high prevalence of poor growth in the cohort (11). There were no infants reported to be receiving non-study multivitamins at 1 month and only 2 infants (both in the placebo group) at 5 months.

### Vitamin B3, Vitamer and Tryptophan Concentrations in Human Milk

**Figure 1:**
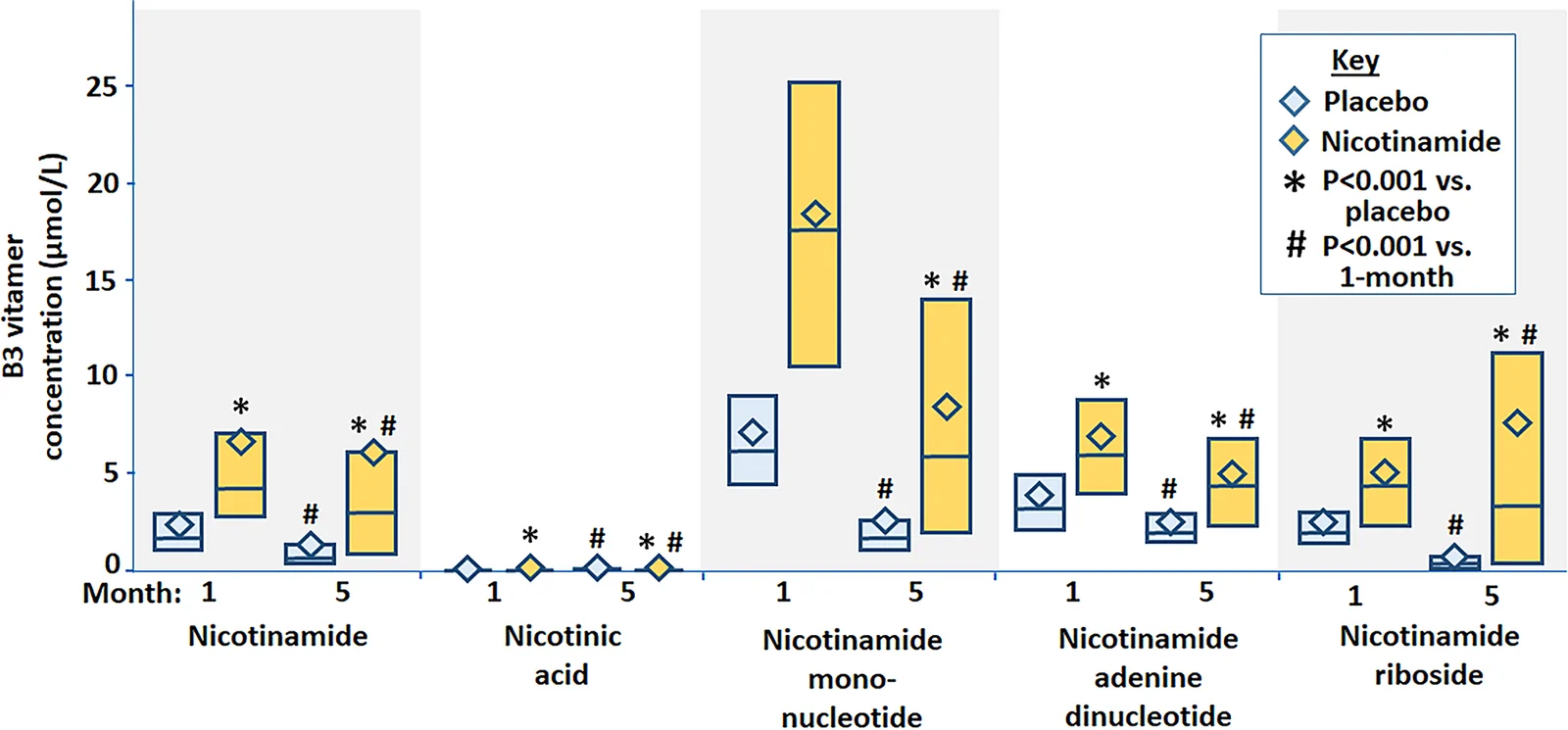
Boxplots of breastmilk vitamin B3 and vitamer concentrations by intervention group and time point. Each box represents the interquartile range 25^th^-75^th^ percentiles, with the central line in each box representing the median and the diamond representing the mean. Participant numbers: 1 month, placebo N=580, nicotinamide supplementation N=583; 5 months, placebo N=572, nicotinamide supplementation N=564.

**Figure 2:**
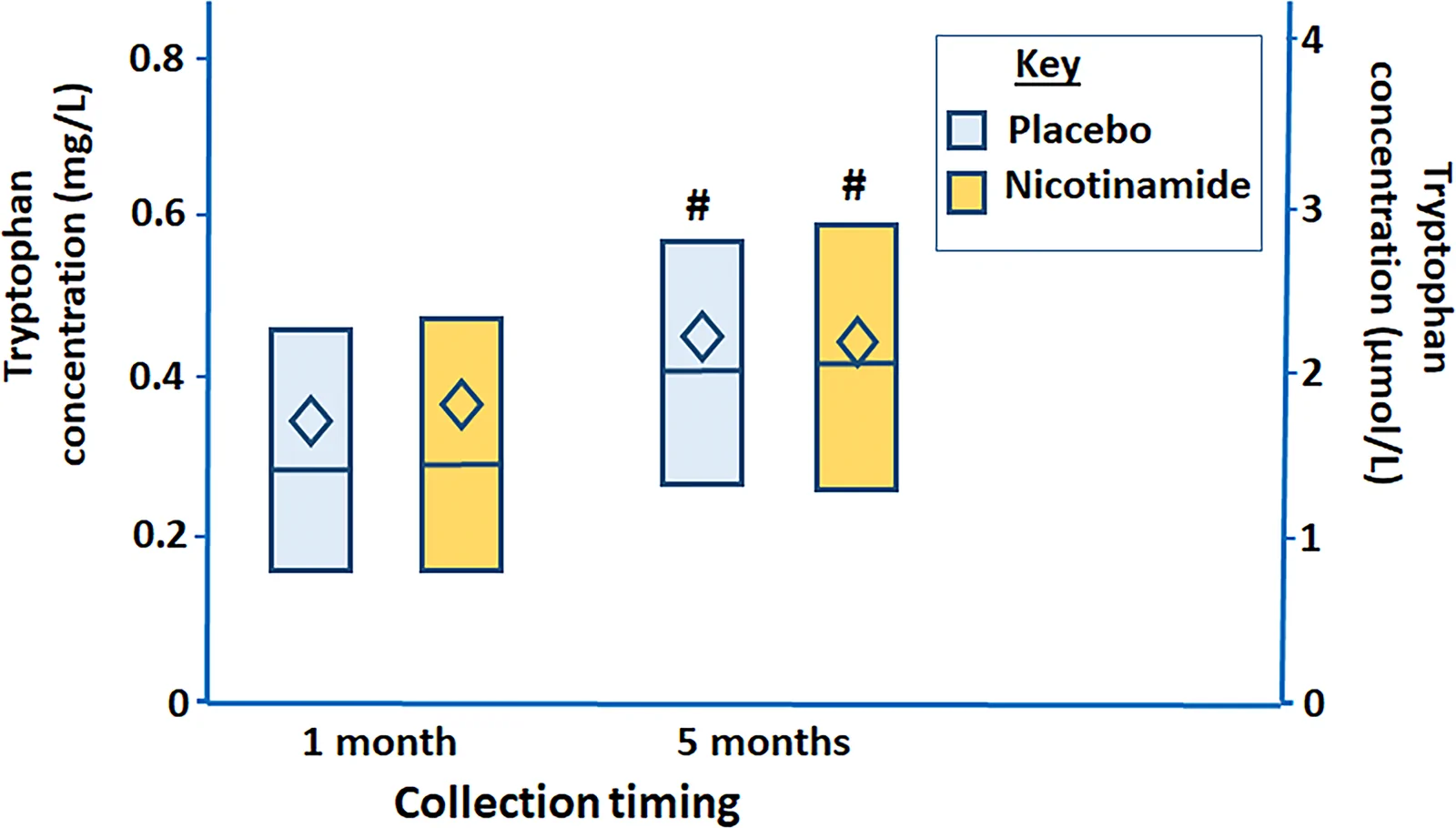
Boxplots of breastmilk tryptophan concentrations by intervention group and time point. Each box represents the interquartile range 25^th^-75^th^ percentiles, with the central line in each box representing the median and the diamond representing the mean. # p<0.0001 vs. 1 month. Participant numbers: 1 month, placebo N=580, nicotinamide supplementation N=583; 5 months, placebo N=572, nicotinamide supplementation N=564.

Human-milk vitamin B3 and vitamer concentrations at lactation months 1 and 5 for mothers treated with placebo or supplemental nicotinamide are displayed as μmol/L in Figure 1 and μg/L in Supplementary Table 2. Mean concentrations of all human milk vitamers were greater in females supplemented with nicotinamide compared to the placebo group (all p<0.001). In the case of nicotinic acid, this difference in human milk was only 20% between treatment groups; for the remainder of vitamers, mothers receiving nicotinamide supplementation had vitamer concentrations 75% to 575% greater than observed in the placebo group. In both supplemented and not supplemented females, the highest concentrations were observed for nicotinamide mononucleotide and the lowest for nicotinic acid. Human milk concentrations of tryptophan are shown in Figure 2 and were not different between mothers who were vs. were not receiving nicotinamide supplementation.

Supplementary Table 3 provides correlations for the individual vitamers between lactation month 1 and 5, which were modestly correlated for both the placebo and nicotinamide groups (Pearson’s *r* values 0.150 to 0.259 for placebo and 0.065 to 0.320 for nicotinamide). Supplementary Table 4 A–C provides correlations between individual vitamer concentrations, overall and for the placebo and nicotinamide groups at 1 and 5 months. At 1 month, with the exception of nicotinic acid, there were high levels of correlation between vitamers in both treatment groups (Pearson’s *r* values 0.224 – 0.532). Nicotinic acid was not significantly correlated with any of the other vitamers, with lower values of Pearson’s *r* (0.012 – 0.232). At 5 months, correlations between vitamer concentrations were higher among those supplemented with nicotinamide compared to placebo, with high Pearson’s *r* values between all vitamers (Pearson’s *r* values 0.234 – 0.717).

### Total Vitamin B3 Concentration in Human Milk by Supplementation Group and in Comparison to Global Reference Ranges

**Figure 3:**
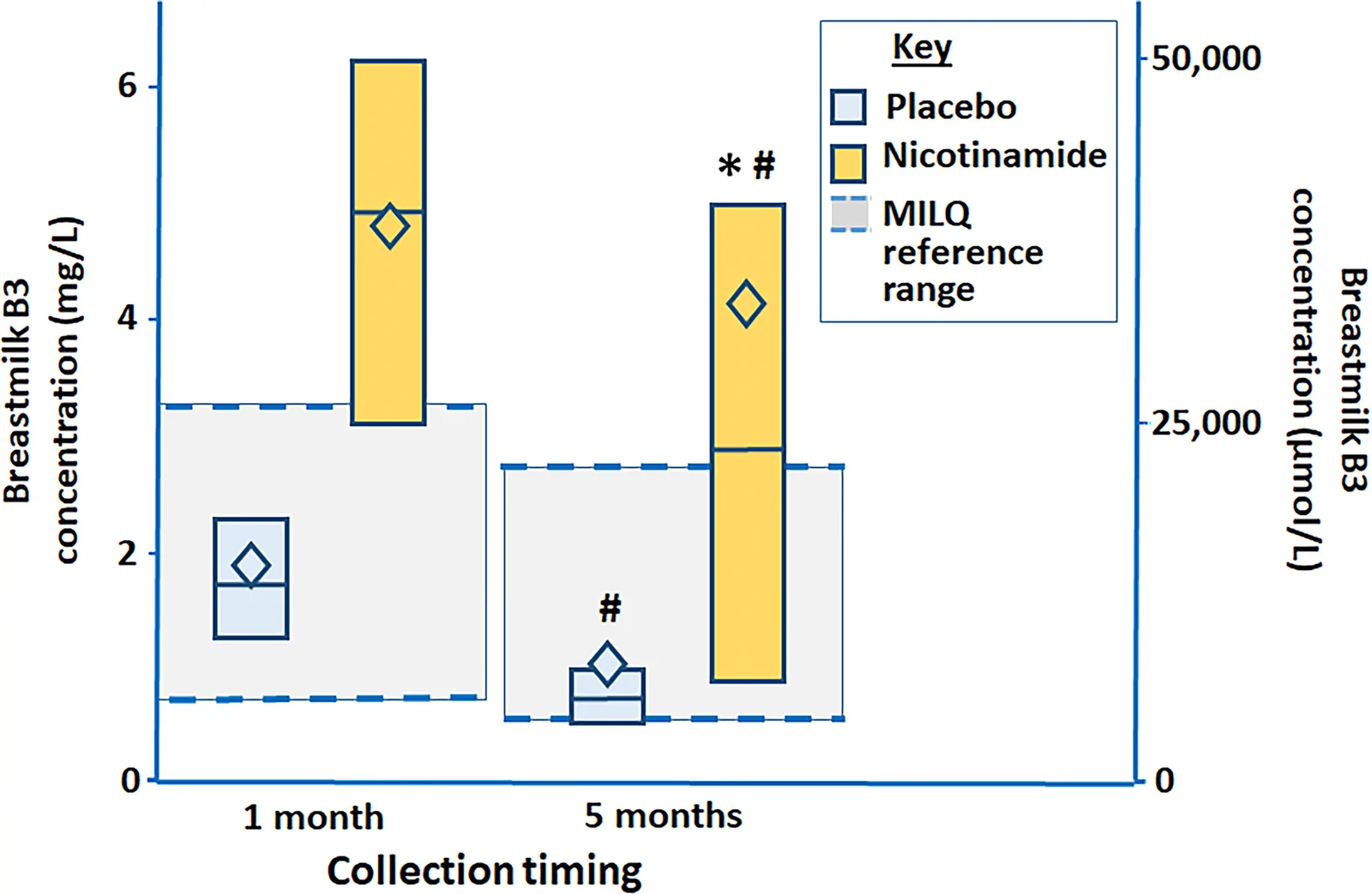
Breastmilk total vitamin B3 concentration at lactation month 1 and 5 months by nicotinamide intervention group and in relation to global reference ranges. Data represent the sum of individual B3 vitamer concentrations from Figure 1. Each box represents the interquartile range 25^th^-75^th^ percentile, with the central line in each box representing the median and the diamond representing the mean. MILQ reference values are from Allen LH, Shahab-Ferdows S, Moore SE, Peerson JM, Kac G, Figueiredo AC, et al. Reference Values for B Vitamins in Human Milk: The Mothers, Infants and Lactation Quality (MILQ) Study. Advances in Nutrition. 2025;19:100500. * p<0.0001 vs. placebo; # p<0.0001 vs. 1 month. Participant numbers: 1 month, placebo N=580, nicotinamide supplementation N=583; 5 months, placebo N=572, nicotinamide supplementation N=564.

Figure 3 displays the combined total vitamin B3 concentrations by supplementation group at lactation month 1 and 5 relative to new global reference ranges (5^th^, 95^th^ percentile) reported from the MILQ study (13). At both time points, concentrations were significantly greater in the nicotinamide-supplemented group compared to the placebo group (median level at month 1=291% greater, month 5=281%; both p<0.0001). Median total vitamin B3 concentrations declined over time in 91% of females in the placebo group (median month 1=1.68 mgl/L; month 5=1.03 mg/L) and in 76% of females in the nicotinamide-supplemented group (month 1=4.9 mg/L; month 5=2.89 μmol/L). The magnitude of the decline was greater in the placebo group (as a percentage of 1-month levels, median= −58%, interquartile range= −70%, −39%) than in the nicotinamide group (median= −41%, interquartile range= −74%, −1%; p<0.0001).

At lactation month 1, only 2.9% of females in the placebo group had human milk total vitamin B3 concentrations below the lower reference limit (5^th^ percentile); however, by lactation month 5, 29% of females without supplementation had concentrations that were below the 5^th^ percentile of the reference range. In comparison, in the nicotinamide supplemented group, only 0.5% of females had human milk total vitamin B3 concentrations below the 5^th^ percentile limit at lactation month 1 and 9.9% at lactation month 5 (both inter-group differences in concentration p<0.0001) (Table 2).

### Human Milk Total Vitamin B3 Concentrations in Relation to Maternal Intervention Adherence and Intervention Dose Timing

Lower maternal adherence to the nicotinamide intervention, assessed by both pill counts and maternal report, was associated with lower human milk total vitamin B3 concentrations at both lactation time points among mothers in the nicotinamide supplementation group (Supplementary Table 5). Human milk concentration of total vitamin B3 was inversely correlated with reported time interval between the last nicotinamide dose and the human milk specimen collection (Supplementary Table 5).

### Human milk Total Vitamin B3 Concentrations in relation to Maternal and Household Characteristics

We next assessed for relationships between total human milk vitamin B3 concentrations and maternal factors including socioeconomic status, parity at enrollment and maternal BMI at 11 months postpartum (Supplementary Table 6). Socioeconomic status (assessed by WAMI (16)) was associated with B3 levels in the placebo group at one but not five months and was not associated in the nicotinamide supplementation group. Associations with parity and maternal BMI were not observed.

### Relationship Between Human milk Total Vitamin B3 Concentrations and Child Anthropometry or Cognitive Outcomes

Supplementary Table 7 displays relationships between total human milk vitamin B3 concentrations and child anthropometry and developmental outcomes. There were no significant associations between human milk total vitamin B3 concentrations and anthropometry outcomes at any time point. Additionally, using the FDR approach to determine p-value significance, there were no associations between human milk total vitamin B3 concentrations and MDAT total or sub-scores scores in either the unadjusted or adjusted regression analyses. This was also true in the sensitivity analysis assessing for differences in outcomes between those with human milk B3 concentrations at 5 months below the 5^th^ percentile reference range compared to those above the 95^th^ percentile (Supplementary Table 8).

### Evaluation for Infant Adverse Events Consuming Elevated Human milk Total Vitamin B3 Concentrations

The potential for infant adverse events with the consumption of elevated human milk vitamin B3 concentrations were considered. At month 1, 1/1177 (0.09%) and at month 5 9/1136 (0.8%) infants consumed human milk with total vitamin B3 concentrations that exceeded at least four times the upper limit of the reference range (Supplementary Tables 9–10). These infants had anthropometry values within two standard deviations of the cohort mean, and differences in reported illness patterns or frequencies around the time that the human milk was consumed were not evident.

For the infant exposed to elevated human milk total vitamin B3 concentrations at month 1, there was one adverse event noted between study entry and age two months (rash at two months). Among the nine infants exposed to elevated human milk total vitamin B3 concentrations at five months, there were adverse events among 4 infants (44%) compared to the entire cohort average of 54% during this same timeframe. Reported illness symptoms included fever, cough, respiratory distress, watery diarrhea and vomiting. One of these infants was hospitalized at 5 months for (pneumonia) and another at 6 months (for gastroenteritis); this hospitalization frequency (22%) was higher than the cohort averages of 1.6% at 5 months and 1% at 6 months.

## DISCUSSION

This study demonstrated that maternal nicotinamide supplementation increases total vitamin B3 and vitamer concentrations throughout lactation, but supplementation was not associated with differences in human-milk tryptophan concentrations. While the majority of females in this study had human-milk total vitamin B3 concentrations remaining above the lower 5^th^ percentile reference limit, by month 5, more than a quarter in the placebo group and close to 10% in the supplemented group had human-milk total vitamin B3 concentrations below the lower global reference limit. Human-milk total vitamin B3 and vitamer concentrations declined among females living in rural Tanzania between lactation months 1 and 5 – a pattern observed in both placebo and nicotinamide-supplemented groups. Finally, human-milk total vitamin B3 concentrations were not associated with future childhood growth or development outcomes by 18 months.

This study follows renewed recognition of the importance of the NAD pathway in energy generation (2) and the recent publication of the MILQ study (13) that provided reference ranges of vitamins and micronutrients in human milk has raised questions about the adequacy of human-milk vitamin B3 concentrations, especially in regions where lactating females consume diets that are at risk of niacin insufficiency. The current study supports the potential for low human-milk concentrations of B3 among many who are not receiving supplementation. The tendency for B3 concentrations to decline over time follows a similar pattern of decline in levels over time from the global reference range (13) and a report from a Malawian study that included nicotinamide supplementation (6). The biological reason for this common decline in human-milk vitamin B3 is unknown, but likely influenced by infant physiological needs as the end of the exclusive breastfeeding period approaches.

In this rural region of north-central Tanzania where maize is locally grown and consumed as a main staple food in conjunction with concerns over niacin bioavailability and limited consumption of niacin-rich foods such as animal, seafood and fortified foods, this study demonstrated that human-milk vitamin B3 concentrations reflecting global reference ranges were achievable from diet alone, with a greater proportion achieved during maternal supplementation with daily nicotinamide. In this study, there were no associations with maternal BMI or parity with differences in human-milk total vitamin B3, and only a weak association at 1 month with an index of socioeconomic status, WAMI, that accounts for household water, assets, income and maternal education. This lack of associations supports that differences we saw between individuals were not from confounding variables, providing evidence for internal validity of the supplementation effect. It should be noted, however, that the maternal ‘cost’ and health implications of providing niacin-adequate human milk on maternal nutritional status, including maternal vitamin B3, vitamers and tryptophan status, in a region with food and nutrition insecurity was unquantified in this study. We were initially surprised that supplying exogenous nicotinamide (and thus removing the need to metabolize tryptophan to niacin) did not alter tryptophan concentrations in human milk—though it remains unclear whether whole-body tryptophan content in mothers changed or whether tryptophan metabolism in the mammary gland compensates through metabolic compartmentalization or regulatory feedback to maintain concentrations in the human milk regardless of whole-body content. Indeed, a prior study found that providing additional tryptophan to lactating women did not alter concentrations in human milk (20).

Although vitamin B3 is an essential micronutrient needed for optimal child health, there were no associations with child growth and development up to 18 months of age. This may be in part due to the fact that the majority of children in this study consumed human milk that exceeded the lower limit of the global reference range, whether their mothers were supplemented with nicotinamide or not. This could potentially reflect a ceiling effect in human milk B3 sufficiency, which limits further gains from supplementation in this context. One key limitation to this interpretation is that while we had robust data on human milk B3 concentrations, we lacked data on the actual volume of human milk children consumed—a key factor required to estimate the total child dose of vitamin B3 that the child consumed. There is potential that mothers with particularly elevated human milk B3 concentrations also had a lower amount of human milk produced daily, with a significant inverse association seen between milk volume and total B3 in this age range in the MILQ study(21). Lower volume could possibly due to dehydration or lower child intake to further stimulate human milk production, either of which may have contributed to lower childhood growth. Finally, in an area of scarcity and food insecurity, there is potential that overall nutrient deficiencies could mask the effect of nicotinamide supplementation (7).

The WHO recommends treating pellagra with 300 mg/day of nicotinamide in divided doses for 3–4 weeks (22). The USDA RDA during lactation is 17 mg daily, and the tolerable upper intake is 35 mg niacin equivalents—meaning that the 250 mg daily of nicotinamide that mothers received over 5.5–6 months was approximately 17-fold above the recommended upper limit and cumulatively above a treatment course for pellagra. We were nevertheless reassured regarding safety, as other pediatric studies had provided nicotinamide doses up to 1000 mg/m^2^ without adverse effects (23). Overall, nicotinamide has fewer side effects than niacin—which itself has been reported to be associated with uncomfortable flushing—but adverse events such as nausea, vomiting and potential liver toxicity can occur with nicotinamide intakes of 3,000 mg/d. In multiple small studies of individuals undergoing hemodialysis, the most common side effects noted from nicotinamide 500–1500 mg/d were diarrhea and thrombocytopenia (24). We did not find a high proportion of diarrhea among infants exposed to high human milk total vitamin B3 concentrations (1 of 9 infants). Additionally, we previously reported similar levels of platelets among infants at age 2, 8 and 18 weeks between placebo- and nicotinamide-treated mother/child dyads in a small subset of participants who had laboratory testing to assess the safety of the intervention (11). Further, other outcomes did not appear different among infants exposed to particularly high human milk concentrations, with the exception of one infant being hospitalized for pneumonia and another for gastroenteritis. Again, there is a caveat that we do not know 1) the actual amount of vitamin B3 these infants consumed as we did not measure the total volume of human milk consumed daily and 2) the infants’ vitamin B3 status, which is difficult to determine from blood testing, as blood levels of niacin and nicotinamide are not a reliable maker of total-body B3 status(25, 26). Also, any potential symptoms (with the exception of hospitalizations) were from retrospective maternal report, which can be inaccurate.

This study benefited from a large well-characterized maternal and child cohort that included data on the maternal and child living environment, maternal and child health and childhood growth and cognition outcomes to 18 months. As mentioned, one important limitation to this study is that human milk volume was unmeasured. As infant dietary vitamin B3 intake is a function of both the human milk vitamin B3 concentration and the human milk volume consumed, we were unable to accurately estimate total infant B3 intake. Future studies should integrate volume measurements (e.g., test weighing, isotope methods) to improve intake-effect modeling. Additionally, we lacked measures in the infant to directly compare to human milk concentration measures and assess the effectiveness of higher human milk B3 concentrations for increasing B3 in the infant—and without such biochemical markers (e.g., urinary markers of B3 metabolites), conclusions about physiological impact remain speculative. Future studies should focus on assessing B3 at the same time points in infants to assess these associations. limitations also included the possibility that infants consumed vitamin B3 from dietary sources other than human milk despite exclusive breastfeeding being reported. Further, new reference ranges of human milk vitamin B3 concentrations are useful to determine where the study concentrations rank in relation to global human milk concentration standards; ideally, however, reference ranges are needed that are associated with positive infant health, growth and development outcomes.

## Conclusion

In this region with maize as the predominant food source, we noted that at infant age 5 months 29% of unsupplemented lactating mothers had human milk total B3 levels below the international reference range. Among females with human milk vitamin B3 concentrations that fall below the 5^th^ percentile reference threshold, maternal nicotinamide supplementation is one option to consider, given higher levels of total B3 levels among those randomized to receive nicotinamide. Further research investigating the health implications of suboptimal human milk vitamin B3 concentrations in this context is needed to guide future public health, maternal and infant care and nutritional recommendations.

## Supplementary Material

Supplementary Material

## Figures and Tables

**Table 1:** Participant characteristics^1^

	Overall N=1173	Placebo N=584	Nicotinamide Supplementation N=593
Mother			
Age, at enrollment (years)	27.7 (6.60)	27.7 (6.9)	27.6 (6.3)
BMI, at 11 months post-partum	21.5 (19.6, 24.1)	21.5 (19.5, 24.2)	21.4 (19.6, 24.0)
Number of children living in household	3.6 (2.2)	3.6 (2.3)	3.6 (2.2)
Education (years)	6.4 (3.0)	6.5 (3.0)	6.3 (3.1)
Income, monthly (US dollars)	12.90 (8.60, 21.50)	12.90 (8.60, 21.50)	12.90 (8.60, 21.50)
WAMI^2^	0.305 (0.129)	0.312 (0.128)	0.299 (0.130)
Exclusive breast feeding duration, months	5 (5, 6)	5 (5, 6)	5 (5, 6)
Infant			
Length			
LAZ entry	−0.799 (1.020)	−0.820 (1.021)	−0.778 (1.019)
LAZ 6 month	−1.159 (1.095)	−1.174 (1.138)	−1.143 (1.049)
LAZ 18 month	−2.020 (0.999)	−2.063 (0.949)	−2.036 (1.048)
Weight			
WAZ entry3	−0.606 (0.980)	−0.600 (0.938)	−0.612 (1.022)
WAZ 6 month	−0.519 (1.086)	−0.550 (1.108)	−0.486 (1.063)
WAZ 18 month	−0.938 (0.963)	−0.936 (0.934)	−0.941 (0.993)

1. Values are expressed as mean (standard deviation) for normally-distributed variables and median (interquartile range) for non-normally-distributed variables.

2. Water, Assets, Maternal education, Income—an index of socioeconomic status derived during the MAL-ED study, including in the area around Haydom; the index is positively associated with socioeconomic status with a range of 0–1 based on participant characteristics for the four components in the name; Psaki SR et al. *Population Health Metrics* 2014, 12:8.

3. Abbreviations: LAZ = length-for-age z-score; WAMI = Water, Assets, Maternal education, Income; WAZ = -for-age z-score.

**Table 2:** Proportion of participants with total vitamin B3 breastmilk concentration below, within and above the 5^th^ and 95^th^ percentiles of the global reference range.^1^

	Overall n, (%)	Placebo n, (%)	Nicotinamide supplementation n, (%)	*P* value
Lactation month 1	N=1163	N=580	N=583	p < 0.0001^1^
Below reference range	20 (1.7%)	17 (2.9%)	3 (0.5%)	
Within reference range	681 (58.6%)	526 (90.7%)	155 (26.6)%	
Above reference range	462 (39.7%)	37 (6.4%)	425 (72.9%)	
				
Lactation month 5	N=1136	N=572	N=564	p < 0.0001^1^
Below reference range	221 (19.5%)	165 (28.8%)	56 (9.9%)	
Within reference range	589 (51.8%)	367 (64.2%)	222 (39.4%)	
Above reference range	326 (28.7%)	40 (7.0%)	286 (50.7%)	

1. Statistical significance assessed via chi square analysis, evaluating whether the distribution of participants between the three categories (below reference range/within reference range/above reference range) differed by intervention group. Reference values reflect the 5^th^ and 95^th^ percentile of breastmilk B3 for 1 and 5 months from Allen LH, Shahab-Ferdows S, Moore SE, Peerson JM, Kac G, Figueiredo AC, et al. Reference Values for B Vitamins in Human Milk: The Mothers, Infants and Lactation Quality (MILQ) Study. Advances in Nutrition. 2025;19:100500.

## Abbreviations:

ELICIT: Early Life Interventions for Childhood Growth and Development in Tanzania
FDR: false discovery rate
HCZ: head-circumference-for-age z-score
LAZ: length-for-age Z-score
LLoQ: lower limit of quantification
MDAT: Malawi Developmental Assessment Tool
NAD: nicotinamide adenine dinucleotide
NE: niacin equivalents
WAMI: water and sanitation, assets, maternal education and household income
WAZ: weight-for-age Z-score
