# Supplementary material for: Human Milk Vitamin B3 (Niacin and Vitamer) Concentrations: Secondary Analysis of a Randomized, Placebo-Controlled Trial of Maternal Nicotinamide Supplementation in Rural Tanzania
Source: Am J Clin Nutr. Author manuscript; Available in PMC 2026 Sep 9. (PMC13555522; doi:10.1016/j.ajcnut.2026.101397)
Supplement: Supplementary file 1 [file NIHMS2192349-supplement-Supplementary_Material.docx]

Human Milk Vitamin B3 (Niacin and Vitamer) Concentrations: Secondary Analysis of a Randomized, Placebo-Controlled Trial of Maternal Nicotinamide Supplementation in Rural Tanzania

DeBoer MD *et al*.

SUPPLEMENTARY FIGURE & TABLES

Supplementary Figure 1: Flow chart for study recruitment, randomization to placebo vs. nicotinamide supplementation and human breastmilk B3 concentrations available for analysis.

Supplementary Table 1: Liquid chromatography-mass chromatography assay characteristics: standard curve range, signal-to-noise ratio, and inter-assay coefficient of variation.^1^

| vitamer | Standard curve range (μg/L) | Signal-to-noise ratio | Inter-assay coefficient of variation |
| --- | --- | --- | --- |
| nicotinamide | 4.0 - 2000 | 843 | 1.7% |
| nicotinic acid | 0.5 - 250 | 50 | 14.5% |
| nicotinamide adenine dinucleotide | 15 - 7500 |  |  |
| nicotinamide mono nucleotide | 6.0 - 3000 | 1187 | 11.5% |
| nicotinamide riboside | 4.0 - 2000 | 7527 | 14.5%. |

1. Values below the standard curve range (considered lower limit of quantification, LLoQ) were included in calculations as being just below the LLoQ, while levels above this range were computed as measured.

Supplementary Table 2: Mean (95% CI) of human milk total vitamin B3, vitamers and tryptophan concentrations (in μg/L).^1^

|  | Lactation month 1 |  |  | Lactation month 5 |  |  |
| --- | --- | --- | --- | --- | --- | --- |
| Human milk analyte | Placebo  N=580 | Nicotinamide Supplementation  N=583 | P value | Placebo  N=572 | Nicotinamide Supplementation  N=564 | P value |
| Nicotinamide | 224.5 (210.6, 239.8) | 574.8 (536.2, 616.1) | <0.001 | 107.6 (98.1, 117.9) | 334.5 (298.4, 375.1) | <0.001 |
| Nicotinic acid | 4.18 (3.67, 4.75) | 5.62 (5.00, 6.30) | <0.001 | 5.62 (5.13, 6.15) | 7.78 (6.95, 8.72) | <0.001 |
| Nicotinamide adenine dinucleotide | 2647 (2507, 2786) | 4680 (4458, 4903) | <0.001 | 1419 (1349, 1494) | 2645 (2490, 2809) | <0.001 |
| Nicotinamide mononucleotide | 2351 (2238, 2466) | 6149 (5883, 6415) | <0.001 | 620.8 (578.5, 666.3) | 1769.8 (1618.1, 1935.8) | <0.001 |
| Nicotinamide riboside | 270.4 (249.6, 295.9) | 1038.8 (940.7, 1147.2) | <0.001 | 161.4 (149.9, 173.8) | 728.1 (638.0, 831.1) | <0.001 |
|  |  |  |  |  |  |  |
| Tryptophan | 275.9 (259.0, 293.8) | 276.0 (257.7, 295.6) | 0.824 | 389.9 (371.9, 408.7) | 383.0 (364.1, 402.9) | 0.800 |

1. Non-normally-distributed variables, mean (95% CI) were calculated from log-transformed data, and back-transformed values reported. On an individual basis (per participant, per timepoint).

Supplementary Table 3: Correlation for each vitamer between 1 and 5 months—overall and by intervention group (placebo, nicotinamide).

|  | Overall  N=1115 |  |  | Placebo  N=553 |  |  | Nicotinamide  N=562 |  |
| --- | --- | --- | --- | --- | --- | --- | --- | --- |
|  | Pearson’s R | P value |  | Pearson’s R | P value |  | Pearson’s R | P value |
| Nicotinamide | 0.370 | <0.0001 |  | 0.259 | <0.0001 |  | 0.172 | <0.0001 |
| Nicotinic acid | 0.145 | 0.001 |  | 0.174 | 0.006 |  | 0.065 | 0.306 |
| Nicotinamide adenine dinucleotide | 0.343 | <0.0001 |  | 0.201 | <0.0001 |  | 0.213 | <0.0001 |
| Nicotinamide mononucleotide | 0.471 | <0.0001 |  | 0.150 | <0.001 |  | 0.320 | <0.0001 |
| Nicotinamide riboside | 0.455 | <0.0001 |  | 0.245 | <0.0001 |  | 0.286 | <0.0001 |
| B3 | 0.505 | <0.0001 |  | 0.161 | <0.0001 |  | 0.301 | <0.0001 |
|  |  |  |  |  |  |  |  |  |
| Tryptophan | 0.148 | <0.0001 |  | 0.089 | 0.035 |  | 0.198 | <0.0001 |

Supplementary Table 4: Correlation between vitamers at 1 and 5 months—overall and by intervention group (placebo, nicotinamide).

A. **Overall**

|  | Nicotinamide | Nicotinic acid | NAD | NM | NR | Total B3 | Tryptophan |
| --- | --- | --- | --- | --- | --- | --- | --- |
| 1 month  (N=1163) |  |  |  |  |  |  |  |
| Nicotinamide |  | 0.278 p≤0.0001 | 0.444 p≤0.0001 | 0.447 p≤0.0001 | 0.448 p≤0.0001 | 0.740 p≤0.0001 | 0.208 p≤0.0001 |
| Nicotinic acid | 0.278 p≤0.0001 |  | 0.257 p≤0.0001 | 0.154 p<0.001 | 0.141 p=0.001 | 0.243 p≤0.0001 | 0.149 p<0.001 |
| NAD | 0.444 p≤0.0001 | 0.257 p≤0.0001 |  | 0.497 p≤0.0001 | 0.396 p≤0.0001 | 0.658 p≤0.0001 | -0.037 p=0.208 |
| Nicotinamide  mononucleotide | 0.447 p≤0.0001 | 0.154 p<0.001 | 0.497 p≤0.0001 |  | 0.637 p≤0.0001 | 0.846 p≤0.0001 | -0.005 p=0.856 |
| Nicotinamide  riboside | 0.448 p≤0.0001 | 0.141 p=0.001 | 0.396 p≤0.0001 | 0.637 p≤0.0001 |  | 0.753 p≤0.0001 | -0.052 p=0.080 |
| Total B3 | 0.740 p≤0.0001 | 0.243 p≤0.0001 | 0.658 p≤0.0001 | 0.846 p≤0.0001 | 0.753 p≤0.0001 |  | 0.065 p=0.028 |
| Tryptophan | 0.208 p≤0.0001 | 0.149 p<0.001 | -0.037 p=0.208 | -0.005 p=0.856 | -0.052 p=0.080 | 0.065 p=0.028 |  |
| 5 month  (N=1136) |  |  |  |  |  |  |  |
| Nicotinamide |  | 0.382 p≤0.0001 | 0.730 p≤0.0001 | 0.672 p≤0.0001 | 0.711 p≤0.0001 | 0.896 p≤0.0001 | 0.120 p≤0.0001 |
| Nicotinic acid | 0.382 p≤0.0001 |  | 0.269 p≤0.0001 | 0.190 p≤0.0001 | 0.233 p≤0.0001 | 0.333 p≤0.0001 | 0.081 p=0.021 |
| NAD | 0.730 p≤0.0001 | 0.269 p≤0.0001 |  | 0.738 p≤0.0001 | 0.653 p≤0.0001 | 0.853 p≤0.0001 | 0.048 p=0.107 |
| Nicotinamide  mononucleotide | 0.672 p≤0.0001 | 0.190 p≤0.0001 | 0.738 p≤0.0001 |  | 0.688 p≤0.0001 | 0.853 p≤0.0001 | 0.021 p=0.476 |
| Nicotinamide  riboside | 0.711 p≤0.0001 | 0.233 p≤0.0001 | 0.653 p≤0.0001 | 0.688 p≤0.0001 |  | 0.857 p≤0.0001 | 0.039 p=0.193 |
| Total B3 | 0.896 p≤0.0001 | 0.333 p≤0.0001 | 0.853 p≤0.0001 | 0.853 p≤0.0001 | 0.857 p≤0.0001 |  | 0.069 p=0.021 |
| Tryptophan | 0.120 p≤0.0001 | 0.081 p=0.021 | 0.048 p=0.107 | 0.021 p=0.476 | 0.039 p=0.193 | 0.069 p=0.021 |  |

Abbreviations: NAD = Nicotinamide adenine dinucleotide; NM = Nicotinamide mononucleotide; NR = Nicotinamide riboside

A. **Placebo**

|  | Nicotinamide | Nicotinic acid | NAD | NM | NR | Total B3 | Tryptophan |
| --- | --- | --- | --- | --- | --- | --- | --- |
| 1 month  (N=580) |  |  |  |  |  |  |  |
| Nicotinamide |  | 0.232 p≤0.0001 | 0.349 p≤0.0001 | 0.226 p≤0.0001 | 0.224 p≤0.0001 | 0.621 p≤0.0001 | 0.271 p≤0.0001 |
| Nicotinic acid | 0.232 p≤0.0001 |  | 0.144 p=0.015 | 0.041 p=0.488 | 0.012 p=0.840 | 0.176 p=0.003 | 0.177 p=0.003 |
| NAD | 0.349 p≤0.0001 | 0.144 p=0.015 |  | 0.339 p≤0.0001 | 0.238 p≤0.0001 | 0.661 p≤0.0001 | -0.001 p=0.984 |
| Nicotinamide mononucleotide | 0.226 p≤0.0001 | 0.041 p=0.488 | 0.339 p≤0.0001 |  | 0.399 p≤0.0001 | 0.774 p≤0.0001 | 0.105 p=0.012 |
| Nicotinamide riboside | 0.224 p≤0.0001 | 0.012 p=0.840 | 0.238 p≤0.0001 | 0.399 p≤0.0001 |  | 0.530 p≤0.0001 | 0.049 p=0.255 |
| Total B3 | 0.621 p≤0.0001 | 0.176 p=0.003 | 0.661 p≤0.0001 | 0.774 p≤0.0001 | 0.530 p≤0.0001 |  | 0.168 p≤0.0001 |
| Tryptophan | 0.271 p≤0.0001 | 0.177 p=0.003 | -0.001 p=0.984 | 0.105 p=0.012 | 0.049 p=0.255 | 0.168 p≤0.0001 |  |
| 5 month  (N=572) |  |  |  |  |  |  |  |
| Nicotinamide |  | 0.224 p≤0.0001 | 0.590 p≤0.0001 | 0.464 p≤0.0001 | 0.524 p≤0.0001 | 0.835 p≤0.0001 | 0.135 p=0.001 |
| Nicotinic acid | 0.224 p≤0.0001 |  | 0.060 p=0.225 | -0.089 p=0.076 | -0.064 p=0.199 | 0.087 p=0.079 | 0.048 p=0.330 |
| NAD | 0.590 p≤0.0001 | 0.060 p=0.225 |  | 0.616 p≤0.0001 | 0.410 p≤0.0001 | 0.812 p≤0.0001 | 0.024 p=0.566 |
| Nicotinamide  mononucleotide | 0.464 p≤0.0001 | -0.089 p=0.076 | 0.616 p≤0.0001 |  | 0.429 p≤0.0001 | 0.766 p≤0.0001 | -0.005 p=0.903 |
| Nicotinamide  riboside | 0.524 p≤0.0001 | -0.064 p=0.199 | 0.410 p≤0.0001 | 0.429 p≤0.0001 |  | 0.667 p≤0.0001 | 0.098 p=0.019 |
| Total B3 | 0.835 p≤0.0001 | 0.087 p=0.079 | 0.812 p≤0.0001 | 0.766 p≤0.0001 | 0.667 p≤0.0001 |  | 0.088 p=0.036 |
| Tryptophan | 0.135  p=0.001 | 0.048 p=0.330 | 0.024 p=0.566 | -0.005 p=0.903 | 0.098 p=0.019 | 0.088 p=0.036 |  |

Abbreviations: NAD = Nicotinamide adenine dinucleotide; NM = Nicotinamide mononucleotide; NR = Nicotinamide riboside

B. **Nicotinamide intervention**

|  | Nicotinamide | Nicotinic acid | NAD | NM | NR | Total B3 | Tryptophan |
| --- | --- | --- | --- | --- | --- | --- | --- |
| 1 month  (N=583) |  |  |  |  |  |  |  |
| Nicotinamide |  | 0.232 p≤0.0001 | 0.290 p≤0.0001 | 0.229 p≤0.0001 | 0.287 p≤0.0001 | 0.644 p≤0.0001 | 0.211 p≤0.0001 |
| Nicotinic acid | 0.232 p≤0.0001 |  | 0.261 p≤0.0001 | 0.087 p=0.130 | 0.112 p=0.051 | 0.201 p<0.001 | 0.116 p=0.044 |
| NAD | 0.290 p≤0.0001 | 0.261 p≤0.0001 |  | 0.351 p≤0.0001 | 0.243 p≤0.0001 | 0.527 p≤0.0001 | -0.064 p=0.122 |
| Nicotinamide  mononucleotide | 0.229 p≤0.0001 | 0.087 p=0.130 | 0.351 p≤0.0001 |  | 0.532 p≤0.0001 | 0.780 p≤0.0001 | -0.051 p=0.223 |
| Nicotinamide  riboside | 0.287 p≤0.0001 | 0.112 p=0.051 | 0.243 p≤0.0001 | 0.532 p≤0.0001 |  | 0.718 p≤0.0001 | -0.140 p=0.001 |
| Total B3 | 0.644 p≤0.0001 | 0.201 p<0.001 | 0.527 p≤0.0001 | 0.780 p≤0.0001 | 0.718 p≤0.0001 |  | 0.025 p=0.540 |
| Tryptophan | 0.211 p≤0.0001 | 0.116 p=0.044 | -0.064 p=0.122 | -0.051 p=0.223 | -0.140 p=0.001 | 0.025 p=0.540 |  |
| 5 month  (N=564) |  |  |  |  |  |  |  |
| Nicotinamide |  | 0.436 p≤0.0001 | 0.734 p≤0.0001 | 0.676 p≤0.0001 | 0.707 p≤0.0001 | 0.906 p≤0.0001 | 0.142 p=0.001 |
| Nicotinic acid | 0.436 p≤0.0001 |  | 0.318 p≤0.0001 | 0.237 p≤0.0001 | 0.282 p≤0.0001 | 0.406 p≤0.0001 | 0.113 p=0.025 |
| NAD | 0.734 p≤0.0001 | 0.318 p≤0.0001 |  | 0.717 p≤0.0001 | 0.656 p≤0.0001 | 0.837 p≤0.0001 | 0.089 p=0.035 |
| Nicotinamide  mononucleotide | 0.676 p≤0.0001 | 0.237 p≤0.0001 | 0.717 p≤0.0001 |  | 0.675 p≤0.0001 | 0.834 p≤0.0001 | 0.056 p=0.185 |
| Nicotinamide  riboside | 0.707 p≤0.0001 | 0.282 p≤0.0001 | 0.656 p≤0.0001 | 0.675 p≤0.0001 |  | 0.860 p≤0.0001 | 0.032 p=0.445 |
| Total B3 | 0.906 p≤0.0001 | 0.406 p≤0.0001 | 0.837 p≤0.0001 | 0.834 p≤0.0001 | 0.860 p≤0.0001 |  | 0.093 p=0.027 |
| Tryptophan | 0.142  p=0.001 | 0.113 p=0.025 | 0.089 p=0.035 | 0.056 p=0.185 | 0.032 p=0.445 | 0.093 p=0.027 |  |

Abbreviations: NAD = Nicotinamide adenine dinucleotide; NM = Nicotinamide mononucleotide; NR = Nicotinamide riboside

Supplementary Table 5: Linear regression assessing relationship between nicotinamide pill count amount, maternal report of missed doses, and time-since-last dose (all as independent variables assessed separately) and human milk total vitamin B3 concentration (dependent variable). Analysis assessed in nicotinamide supplementation group only.

| Outcome | Predictor | Estimate (95% CI)^1^ | p value |
| --- | --- | --- | --- |
| Human milk total vitamin B3 at lactation month 1  (N=583) | Pill count at 2 months  (quantified as the percentage of pills taken) | 0.592 (0.109, 1.076) | 0.017 |
|  | Maternal self-report of missed doses at 1 month  (quantified as the number of pills missed in the past 7 days) | -0.093 (-0.146, -0.040) | <0.001 |
|  | Time lapse between the last intervention dose and time of the human milk specimen collection (quantified as number of hours) | -0.00013 (-0.00020, -0.00005) | 0.001 |
| Human milk total vitamin B3 at lactation month 5  (N=580) | Pill count at 4 months  (quantified as the percentage of pills taken) | 0.760 (0.249, 1.271) | 0.004 |
|  | Maternal self-report of missed doses at 5 months  (quantified as the number of pills missed in the past 7 days) | -0.120 (-0.178, -0.061) | <0.0001 |

1. Estimate is of standardized total vitamin B3 concentration; thus each estimate of 1.0 represents one standard deviation of human milk total vitamin B3 concentration per individual predictor unit (i.e. percentage of pills taken, number of pills missed or hour since last dose taken).

Supplementary Table 6: Human milk total vitamin B3 concentrations according to socioeconomic status, parity at enrollment and maternal BMI at 11 months postpartum.^1^

|  | 1 month |  |  | 5 months | p value |
| --- | --- | --- | --- | --- | --- |
|  | Placebo  (N=580) | Nicotinamide Supplementation  (N=583) |  | Placebo  (N=572) | Nicotinamide Supplementation  (N=564) |
| Socioeconomic status |  |  |  |  |  |
| WAMI^2^ lower half | 1651 (1185, 2220) | 4883 (3096, 5953) |  | 688 (525, 941) | 2692 (799, 5380) |
| WAMI upper half | 1687 (1268, 2288) | 4936 (3126, 6420) |  | 729 (530, 9565) | 3056 (932, 5010) |
| Regression estimate | 0.056 (0.001, 0.112) | 0.035 (-0.031, 0.102) |  | 0.047 (-0.008, 0.101) | 0.009 (-0.075, 0.093) |
| p value | 0.047 | 0.296 |  | 0.092 | 0.836 |
| Parity |  |  |  |  |  |
| Firstborn | 1734 (1237, 2021) | 5064 (3293, 6237) |  | 667 (504, 925) | 2311 (888, 5668) |
| Not firstborn | 1675 (1236, 2279) | 4878 (3073, 6237) |  | 723 (521, 956) | 3932 (878, 5011) |
| p value | 0.212 | 0.606 |  | 0.228 | 0.735 |
| BMI, 11 months postpartum |  |  |  |  |  |
| Underweight | 1564 (1199, 2140) | 4802 (3752, 6335) |  | 674 (500, 972) | 3180 (950, 5783) |
| Normal weight | 1654 (1198, 2163) | 4986 (3052, 6326) |  | 704 (496, 947) | 3095 (893, 5292) |
| Overweight | 1952 (1376, 2433) | 5052 (3253, 6255) |  | 750 (591, 952) | 2163 (815, 4120) |
| Obese | 1733 (1374, 2604) | 4394 (2954, 4929) |  | 820 (612, 910) | 1915 (750, 4338) |
| Regression estimate | 0.056 (-0.000, 0.112) | -0.035 (-0.104, 0.034) |  | 0.039 (-0.016, 0.093) | -0.072 (-0.159, 0.015) |
| p value | 0.051 | 0.317 |  | 0.174 | 0.106 |

1. Data presented are median (interquartile range). P values for WAMI and BMI are from linear regression using standardized values of WAMI and BMI as the independent variables and standardized values of human milk total vitamin B3 concentration as the dependent variable. For parity, p-values are from t-tests for normally distributed values and t-tests on log-transformed data for non-normally distributed values.

2. Water, Assets, Maternal education, Income—an index of socioeconomic status derived during the MAL-ED study, including in the area around Haydom; the index is positively associated with SES with a range of 0-1 based on participant characteristics for the four components in the name; Psaki SR et al. *Population Health Metrics* 2014, 12:8.

Supplementary Table 7: Relationship between total human milk vitamin B3 concentrations at lactation month 1 and 5 and childhood anthropometry or cognitive development outcomes to 18-months. Data shown represent regression-derived beta coefficients from cohort-standardized total B3 concentrations (with 95% confidence interval).

|  | 1 Month (N=1148)^1^ | | 5 Month (N=1132)^1^ | |
| --- | --- | --- | --- | --- |
|  | Unadjusted | Adjusted^2^ | Unadjusted | Adjusted^2^ |
| Child Anthropometry^3^ | | | | |
| 3-month |  |  |  |  |
| LAZ | 0.037 (-0.021, 0.096) p=0.207 | 0.019 (-0.030, 0.068) p=0.448 | NA | NA |
| WAZ | 0.011 (-0.047, 0.069) p=0.715 | 0.020 (-0.030, 0.070) p=0.432 | NA | NA |
| HCZ | -0.003 (-0.049, 0.043) p= 0.912 | -0.002 (-0.048, 0.044) p= 0.936 | NA | NA |
| 6-month |  |  |  |  |
| LAZ | -0.030 (-0.088, 0.028) p=0.307 | -0.040 (-0.092, 0.012) p=0.134 | 0.012 (-0.046, 0.070) p=0.679 | -0.003 (-0.055, 0.050) p=0.925 |
| WAZ | -0.009 (-0.067, 0.049) p=0.764 | 0.003 (-0.050, 0.056) p=0.907 | -0.005 (-0.063, 0.054) p=0.871 | -0.001 (-0.054, 0.053) p=0.980 |
| HCZ | 0.027 (-0.022, 0.076) p= 0.277 | 0.028 (-0.021, 0.077) p= 0.262 | -0.025 (-0.073, 0.024) p= 0.314 | -0.026 (-0.075, 0.022) p= 0.283 |
| 9-month |  |  |  |  |
| LAZ | -0.009 (-0.069, 0.050) p=0.762 | -0.019 (-0.072, 0.034) p=0.477 | -0.009 (-0.050, 0.071) p=0.732 | 0.000 (-0.054, 0.053) p=0.992 |
| WAZ | -0.031 (-0.090, 0.029) p=0.309 | -0.021 (-0.077, 0.035) p=0.462 | -0.045 (-0.105, 0.015) p=0.145 | -0.037 (-0.093, 0.020) p=0.200 |
| HCZ | 0.016 (-0.036, 0.068) p= 0.556 | 0.020 (-0.032, 0.071) p= 0.455 | 0.000 (-0.052, 0.052) p= 0.989 | 0.001 (-0.051, 0.052) p= 0.977 |
| 18-month |  |  |  |  |
| LAZ | 0.007 (-0.053, 0.066) p=0.827 | -0.002 (-0.055, 0.052) p=0.954 | 0.033 (-0.026, 0.092) p=0.277 | 0.016 (-0.037, 0.070) p=0.550 |
| WAZ | -0.019 (-0.079, 0.040) p=0.529 | -0.009 (-0.065, 0.048) p=0.765 | -0.008 (-0.067, 0.051) p=0.795 | -0.004 (-0.059, 0.052) p=0.898 |
| HCZ | 0.003 (-0.046, 0.053) p= 0.893 | 0.004 (-0.046, 0.053) p= 0.889 | 0.018 (-0.031, 0.067) p= 0.475 | 0.017 (-0.032, 0.066) p= 0.508 |
| Cognitive development^4^ (18 month)^3^ | | | | |
| MDAT Overall  score | -0.058 (-0.119, -0.004) p= 0.065 | -0.063 (-0.123, -0.019) p= 0.043 | -0.028 (-0.086, 0.030) p= 0.348 | -0.033 (-0.091, 0.024) p= 0.257 |
| MDAT Fine  Motor score | -0.013 (-0.072, 0.046) p= 0.660 | -0.014 (-0.073, 0.045) p= 0.636 | -0.040 (-0.098, 0.017) p= 0.171 | -0.041 (-0.098, 0.017) p= 0.163 |
| MDAT Gross  Motor score | -0.041 (-0.100, 0.019) p= 0.180 | -0.043 (-0.101, 0.016) p= 0.156 | -0.060 (-0.121, 0.000) p= 0.049 | -0.062 (-0.121, -0.002) p= 0.043 |
| MDAT  Language score | -0.026 (-0.086, 0.034) p= 0.396 | -0.027 (-0.086, 0.032) p= 0.370 | 0.005 (-0.054, 0.063) p= 0.878 | 0.000 (-0.057, 0.058) p= 0.996 |
| MDAT Social  score | -0.070 (-0.131, -0.008) p= 0.026 | -0.074 (-0.134, -0.032) p= 0.017 | 0.001 (-0.057, 0.059) p= 0.970 | -0.005 (-0.062, 0.052) p= 0.876 |

1. Number represents number of participants included in at least one analysis. Among all participants with any human milk B3 concentrations, availability of data for this analysis (and number of participants missing data) from analysis is as follows:

|  | Baseline | 3 | 6 | 9 | 18 |
| --- | --- | --- | --- | --- | --- |
| WAMI | 1173 (0) |  |  |  |  |
| sex | 1173 (0) |  |  |  |  |
| LAZ | 1167 (6) | 1135 (38) | 1131 (42) | 1073 (100) | 1084 (89) |
| WAZ | 1167 (6) | 1143 (30) | 1130 (43) | 1083 (90) | 1090 (83) |
| HCZ | 1167 (6) | 1141 (32) | 1129 (44) | 1076 (97) | 1088 (85) |
| MDAT |  |  |  |  | 1032 (141) |

2. Analyses adjusted for sex, SES, baseline value (for anthropometry measures).

3. Given the number of tests assessed simultaneously, we used a hierarchical false discovery rate (FDR) approach to determine statistically-significant p values. Using this approach, the initial p value cut off to be considered statistically significant for anthropometry outcomes was 0.017 and for cognitive development was 0.010. Using this approach, no relationships were statistically significant.

4. Malawi Developmental Assessment Tool is composed of four separate domains (Fine Motor, Gross Motor, Language and Social) which are summed for an overall score. All scores have been standardized for the full cohort to produce overall mean of 0 with standard deviation of 1.0.

Abbreviations: HCZ = head circumference z-score; LAZ = length-for-age z-score; MDAT = Malawi Developmental Assessment Tool; WAZ = weight-for-age z-score

Supplementary Table 8: Regression analysis assessing children whose human milk B3 concentrations at 5 months were below the reference range 5^th^ percentile vs. greater than 95^th^ percentile.

|  | 5 Month (N=1132)^1^ | |
| --- | --- | --- |
|  | Unadjusted | Adjusted^2^ |
| 6-month |  |  |
| LAZ | 0.100 (-0.089, 0.288) p= 0.300 | 0.018 (-0.149, 0.185) p= 0.830 |
| WAZ | 0.074 (-0.121, 0.269) p= 0.455 | 0.012 (-0.159, 0.182) p= 0.894 |
| HCZ | 0.090 (-0.086, 0.267) p= 0.315 | 0.013 (-0.143, 0.170) p= 0.866 |
| 9-month |  |  |
| LAZ | 0.043 (-0.145, 0.231) p= 0.650 | 0.011 (-0.164, 0.185) p= 0.906 |
| WAZ | -0.093 (-0.287, 0.101) p= 0.346 | -0.101 (-0.284, 0.082) p= 0.279 |
| HCZ | 0.022 (-0.152, 0.197) p= 0.801 | -0.004 (-0.166, 0.158) p= 0.960 |
| 18-month |  |  |
| LAZ | -0.093 (-0.277, 0.091) p= 0.320 | -0.123 (-0.296, 0.050) p= 0.164 |
| WAZ | 0.001 (-0.188, 0.191) p= 0.989 | -0.014 (-0.194, 0.166) p= 0.878 |
| HCZ | 0.078 (-0.089, 0.245) p= 0.360 | 0.051 (-0.108, 0.210) p= 0.533 |
| Cognitive development^4^ (18 month)^3^ | | |
| MDAT Overall  score | -0.107 (-0.254, 0.039) p= 0.151 | -0.120 (-0.265, 0.026) p= 0.107 |
| MDAT Fine  Motor score | -0.155 (-0.381, 0.071) p= 0.177 | -0.155 (-0.381, 0.070) p= 0.177 |
| MDAT Gross  Motor score | -0.082 (-0.206, 0.041) p= 0.189 | -0.091 (-0.213, 0.032) p= 0.146 |
| MDAT  Language score | -0.045 (-0.232, 0.143) p= 0.640 | -0.058 (-0.245, 0.129) p= 0.546 |
| MDAT Social  score | -0.070 (-0.183, 0.042) p= 0.220 | -0.080 (-0.191, 0.032) p= 0.160 |

1. Number represents number of participants included in at least one analysis.

2. Analyses adjusted for sex, SES, baseline value (for anthropometry measures).

3. Given the number of tests assessed simultaneously, we used a hierarchical false discovery rate (FDR) approach to determine statistically-significant p values. Using this approach, the initial p value cut off to be considered statistically significant for anthropometry outcomes was 0.017 and for cognitive development was 0.010. Using this approach, no relationships were statistically significant.

4. Malawi Developmental Assessment Tool is composed of four separate domains (Fine Motor, Gross Motor, Language and Social) which are summed for an overall score. All scores have been standardized for the full cohort to produce overall mean of 0 with standard deviation of 1.0.

Abbreviations: HCZ = head circumference z-score; LAZ = length-for-age z-score; MDAT = Malawi Developmental Assessment Tool; WAZ = weight-for-age z-score

Supplementary Table 9: Anthropometry, hospitalization and illness data for the one participant whose mother’s human milk at 1 month had a total vitamin B3 concentration more than 4 times the upper limit of the normal range, along with total cohort values.

| 1 month: Children consuming human milk with B3 concentrations ≥4 times the upper reference range threshold (>13,136 μg/L) | | |
| --- | --- | --- |
|  | Child 1 | Total cohort mean (N=1173) |
| Childhood anthropometry & cognitive development measurements |  |  |
| LAZ 3 months | 0.56 | -0.85 |
| WAZ 3 months | 1.33 | -0.56 |
| HCZ 3 months | 1.06 | -0.26 |
| LAZ 6 months | 0.17 | -1.16 |
| WAZ 6 months | 1.28 | -0.52 |
| HCZ 6 months | 0.30 | -0.30 |
| MDAT z-score | -0.37 | 0.67 |
| Infant medical attention reported over past month |  |  |
| Sought health care, 1 month | 0 | 13.3% |
| Sought health care, 2 months | 0 | 15.2% |
| Hospitalized, 1 month | 0 | 1.0% |
| Hospitalized, 2 months | 0 | 1.1% |
| Infant’s illness symptoms reported over past week |  |  |
| Diarrhea, 1 month | 0 | 2.0% |
| Diarrhea, 2 months | 0 | 3.5%# |
| Cough, 1 month | 0 | 6.8% |
| Cough, 2 months | 0 | 8.8% |
| Fever, 1 month | 0 | 4.9% |
| Fever, 2 months | 0 | 4.5% |
| Adverse events |  |  |
| Adverse event 0-2 months,  mother | 0 | 6.1% |
| Adverse event 0-2 months,  infant | 1^1^ | 21.5% |

1. Adverse events:

Child 1: Child had intermittent rash; did not seek medical attention

Supplementary Table 10: Anthropometry, hospitalization and illness data for nine participants whose mother’s human milk at 5 months had a total vitamin B3 concentration more than 4 times the upper limit of the normal range, along with total cohort values.

|  | 5 months child participants with B3 levels ≥4 times upper limit of reference range (>11,040 μg/L) | | | | | | | | |  |
| --- | --- | --- | --- | --- | --- | --- | --- | --- | --- | --- |
|  | Child 1 | Child 2 | Child 3 | Child 4 | Child 5 | Child 6 | Child 7 | Child 8 | Child 9 | Whole cohort mean  (N=1173) |
| Anthropometry & cognitive development measurements |  |  |  |  |  |  |  |  |  |  |
| LAZ 6 months | -2.03 | -1.52 | -2.05 | 0.73 | -1.61 | -0.21 | -0.73 | -0.15 | 0.5 | -1.16 |
| WAZ 6 months | -0.83 | -0.77 | -1.88 | -1.08 | 0.46 | 1.17 | -0.09 | 1.08 | 1.21 | -0.52 |
| HCZ 6 months | 0.37 | -0.44 | -2.64 | -2.66 | 0.64 | 1.08 | -0.13 | 0.66 | 1.57 | -0.29 |
| LAZ 9 months | -2.12 | LTFU | -1.68 | -0.78 | -2.1 | -1.21 | -2.1 | -0.37 | 0.03 | -1.39 |
| WAZ 9 months | -0.99 | LTFU | -1.52 | -1.05 | 0.38 | NA | -0.21 | 0.13 | 1.13 | -0.58 |
| HCZ 9 months | -0.74 | LTFU | -1.38 | -1.06 | 0.3 | 1.23 | -0.6 | 1.15 | 1.45 | -0.38 |
| MDAT_z-score | 1.48 | LTFU | 0.34 | 1.23 | 1.05 | 0.93 | 0.94 | 1.05 | 1.43 | 0.67 |
| Child’s medical attention reported over past month |  |  |  |  |  |  |  |  |  |  |
| Sought health care, 5 mo | 0 | 0 | 1 | 0 | 0 | 0 | 0 | 0 | 0 | 17.9% |
| Sought health care, 6 mo | 1 | 0 | 0 | 1 | 0 | 0 | 0 | 1 | 0 | 21.0% |
| Hospitalized, 5 mo | 0 | 0 | 1 | 0 | 0 | 0 | 0 | 0 | 0 | 1.6%# |
| Hospitalized, 6 mo | 0 | 0 | 0 | 1 | 0 | 0 | 0 | 0 | 0 | 1.0%## |
| Child’s illness symptoms reported over past week |  |  |  |  |  |  |  |  |  |  |
| Diarrhea, 5 mo | 0 | 0 | 1 | 0 | 0 | 0 | 0 | 0 | 0 | 4.4% |
| Diarrhea, 6 mo | 0 | 0 | 0 | 1 | 0 | 0 | 0 | 0 | 0 | 7.5% |
| Cough, 5 mo | 0 | 0 | 1 | 0 | 0 | 0 | 0 | 0 | 0 | 10.0% |
| Cough, 6 mo | 1 | 0 | 0 | 1 | 0 | 0 | 0 | 1 | 0 | 13.5%# |
| Fever, 5 mo | 0 | 0 | 0 | 0 | 0 | 0 | 0 | 0 | 0 | 5.3% |
| Fever, 6 mo | 1 | 0 | 0 | 1 | 0 | 0 | 0 | 0 | 0 | 7.7% |
| Adverse events |  |  |  |  |  |  |  |  |  |  |
| Adverse event 4-6 mo, mother | 0 | 1* | 0 | 0 | 0 | 0 | 0 | 0 | 0 | 14.3% |
| Adverse event 4-6 mo, child | 1^1^ | 0 | 1^1^ | 1^1^ | 0 | 0 | 0 | 1^1^ | 0 | 53.7% |

Abbreviations: HCZ: Head circumference Z-score; LAZ: Length for age Z-score; LTFU: Lost to follow-up; MDAT: Malawi Developmental Assessment Tool; NA: Not available; WAZ: Weight for age Z-score.

1. Adverse events:

Child 1: Child had cough and fever; received amoxicillin at pharmacy and fully recovered.

Child 2: Mother had low Hb of 11.1; family subsequently moved out of area and was lost to follow-up.

Child 3: Child hospitalized for “fever, cough, difficulty breathing;” diagnosed with pneumonia; condition improved and discharged on amoxicillin.

Child 4: Child hospitalized for cough, fever, watery diarrhea and vomiting; diagnosed with gastroenteritis and dehydration.

Child 8: Child had cough and runny nose; given erythromycin for treatment.

Statistical comparison of proportions of those with elevated human milk B3 with full cohort:

\# p<0.05; ## p<0.01
